# Robust estimation of skin physiological parameters from hyperspectral images using Bayesian neural networks

**DOI:** 10.1117/1.JBO.30.1.016004

**Published:** 2025-01-16

**Authors:** Teo Manojlović, Tadej Tomanič, Ivan Štajduhar, Matija Milanič

**Affiliations:** a University of Rijeka, Faculty of Engineering, Rijeka, Croatia; b University of Rijeka, Center for Artificial Intelligence and Cybersecurity, Rijeka, Croatia; c University of Ljubljana, Faculty of Mathematics and Physics, Ljubljana, Slovenia; d Jozef Stefan Institute, Ljubljana, Slovenia

**Keywords:** hyperspectral imaging, neural networks, diffuse reflectance spectra, inverse adding-doubling

## Abstract

**Significance:**

Machine learning models for the direct extraction of tissue parameters from hyperspectral images have been extensively researched recently, as they represent a faster alternative to the well-known iterative methods such as inverse Monte Carlo and inverse adding-doubling (IAD).

**Aim:**

We aim to develop a Bayesian neural network model for robust prediction of physiological parameters from hyperspectral images.

**Approach:**

We propose a two-component system for extracting physiological parameters from hyperspectral images. First, our system models the relationship between the measured spectra and the tissue parameters as a distribution rather than a point estimate and is thus able to generate multiple possible solutions. Second, the proposed tissue parameters are then refined using the neural network that approximates the biological tissue model.

**Results:**

The proposed model was tested on simulated and *in vivo* data. It outperformed current models with an overall mean absolute error of 0.0141 and can be used as a faster alternative to the IAD algorithm.

**Conclusions:**

Results suggest that Bayesian neural networks coupled with the approximation of a biological tissue model can be used to reliably and accurately extract tissue properties from hyperspectral images on the fly.

## 1 Introduction

Hyperspectral imaging (HSI) is a technique that combines imaging and spectroscopy to measure and collect spatially resolved spectra in a three-dimensional hypercube consisting of two spatial and one spectral dimension.^1^ In medicine, HSI has recently been used as a promising, non-invasive, and cost-effective technique for measuring various tissue properties related to physiology, morphology, or structure.^1^ Based on the idea that specific diseases change the spectra,^2^ several studies have reported on the usability of HSI for surgery, diagnosis, and therapy.^1^

To obtain the relevant tissue properties from medical hyperspectral images, light propagation is usually performed in a specific biological tissue model. The most commonly used method is the inverse Monte Carlo (IMC),^3^ which is considered very accurate, albeit computationally intensive.^4^^,^^5^ One way to reduce the computation time is to use simpler approaches such as the inverse diffusion approximation,^6^ or the Beer–Lambert (BL) law.^5^^,^^7^ However, these methods are generally less accurate. In contrast to the methods mentioned, inverse adding-doubling (IAD)^8^ has proven to provide a good balance between accuracy and computation speed. Although the computation time can be significantly reduced using graphics processing units (GPUs)^9^ or by approximating the forward model using a lookup table,^10^ these models are still not suitable for real-time extraction of tissue properties from hyperspectral images.

As an alternative to the traditional approaches, machine learning (ML) has also been applied to extract physiological properties from hyperspectral images, mainly to reduce computation time. ML models are used either as part of a curve fitting process to approximate a forward model,^11^ or they are used to directly estimate tissue properties from the observed spectra.^12^^,^^13^

However, despite recent advances in ML models for estimating physiological properties from hyperspectral images, they are not yet widely used in clinical practice. Many authors explored different ideas as to why the models suffer from performance degradation when used on *in vivo* data. Chen and Tseng^14^ considered the size and complexity of the training dataset and proposed building the training dataset by verifying that the different sets of optical properties do not result in the same or similar spectra. Alternatively, multiple artificial neural networks (ANNs) can be used,^15^ where the spectra are split into groups based on their shape, which are then used to train individual neural networks. Compared with a single ANN, the average relative error of the reduced scattering coefficient was reduced from 4.1% to 2.9%, whereas the average relative error of the absorption was reduced from 9.5% to 6.1%. Fredriksson et al.^16^ used an ANN for direct estimation of oxygen saturation and hemoglobin concentration by trying to model the noise of the HSI system. They proposed a noise model consisting of measurement noise, residual noise, and color uncertainty. Manojlović et al.^17^ evaluated commonly used ML algorithms for direct extraction of physiological properties of human skin. In this study, it has been shown that the differences in the results of a simulated and an *in vivo* dataset are not only caused by the noise but also by the surface topography. Scarbrough et al.^18^ performed an in-depth analysis of ML model performance by identifying three main types of use errors that can occur when measuring diffuse reflectance spectra: noise, wavelength miscalibrations, and spectral intensity variations. They proposed to incorporate these use errors into the training and test dataset by modifying the signal with a combination of Gaussian noise, rotation, shift, scaling, and compression. Finally, Ezhov et al.^19^ compared two different strategies to create the training dataset. In the first strategy, synthetic data were used to train the ML models, whereas in the second strategy, the ML models were trained on real spectra for which the parameters were obtained using traditional least squares fitting. Results have shown that training the ML models using the second strategy outperforms the ML models trained only on the synthetic data. However, training on real data is not always possible, especially in cases where the tissue model is complex or the number of volunteers from which the spectra were extracted is small.

In this study, we propose a different approach to obtain a more robust model trained on the dataset simulated using adding-doubling (AD). First, we propose to transform the original input signal using random Fourier features (RFFs).^20^ Next, we use a Bayesian neural network (BNN) instead of the commonly used ANN, allowing us to obtain multiple possible solutions. Finally, we introduce an additional neural network that is trained to approximate AD. This component not only allows us to select the parameters for which the spectra generated by the forward model have a better alignment with measured spectra, but it is also possible to immediately recognize spectra for which the model does not provide a satisfactory result and fall back on traditional and more accurate methods.

To summarize, the main contributions of this paper are the following:

1. We propose a random Fourier feature–based Bayesian neural network (RFF + BNN) to model the relationship between the measured spectra and the physiological skin parameters and show that it produces more robust solutions in comparison with the ANN or one-dimensional convolutional neural network (1D-CNN) trained on raw spectra.
2. The proposed model is trained on uniformly sampled simulated spectra with minimum prior knowledge of the actual properties of the observed tissue and without introducing use errors into the training process, making it easily adaptable to different tissue types while also retaining robustness.
3. We propose to use a neural network approximating the forward model (F-NN) to select the best-fitted sampled parameters.
4. The proposed method does not require iterative curve fitting, so it can be easily parallelized on modern GPU architectures.

## 2 Methods

### 2.1 Two-Layer Skin Model

**Fig. 1:**
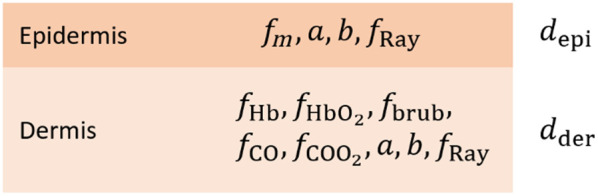
Two-layer skin model consisting of epidermis and dermis with a total of 11 physiological parameters used in our study. Adopted from Tomanič et al.^21^

In this study, we consider a two-layer model of human skin with 11 parameters proposed in the previous work by Tomaničc et al.,^21^ as shown in Fig. 1. To model the propagation of light through the skin model, we use the IAD algorithm,^8^^,^^21^^,^^22^ which proves to be much faster than the commonly used IMC, with the spectra agreement on the fourth decimal place. To apply the IAD to the skin model in Fig. 1, the absorption coefficients for the epidermis ($\mu_{a,\mathrm{epi}}$) and dermis ($\mu_{a,\mathrm{der}}$) as well as the scattering coefficient ($\mu_{s}^{\prime }$), the refractive index (n), and the anisotropy factor (g) must be calculated.

The absorption coefficient of the epidermis is calculated using the following equation:^21^^,^^23^^,^^24^

$$\mu_{a,\mathrm{epi}}=f_{m}\mu_{a,m}+\mu_{a,\text{base}}.$$ (1)

As can be seen, the absorption coefficient of the epidermis is the sum of the melanin absorption coefficient ($\mu_{a,m}$) and the baseline absorption of the bloodless skin ($\mu_{a,\text{base}}$), which is given by the following equations:

$$\mu_{a,m}=6.6\cdot 10^{11}\;{\mathrm{cm}}^{-1}{\left(\frac{\lambda }{\mathrm{nm}}\right)}^{-3.33},$$ (2)

$$\mu_{a,\text{base}}=0.244\;{\mathrm{cm}}^{-1}+85.3\;{\mathrm{cm}}^{-1}\cdot e^{-\frac{\lambda -154\;\mathrm{nm}}{66.2\;\mathrm{nm}}},$$ (3)

where λ is the wavelength in nanometers, and $f_{m}$ is the volume fraction of melanin, as described in Table 2. To calculate the absorption coefficient of the dermis $\mu_{a,\mathrm{der}}$, we use the following equation:^21^^,^^23^

$$\mu_{a,\mathrm{der}}=f_{\mathrm{Hb}}\mu_{a,\mathrm{Hb}}+f_{{\mathrm{HbO}}_{2}}\mu_{a,{\mathrm{HbO}}_{2}}+f_{\text{brub}}\mu_{a,\text{brub}}+f_{\mathrm{CO}}\mu_{a,\mathrm{CO}}+f_{{\mathrm{COO}}_{2}}\mu_{a,{\mathrm{COO}}_{2}}+\mu_{a,\text{base}},$$ (4)

where $f_{\mathrm{Hb}}$, $f_{{\mathrm{HbO}}_{2}}$, $f_{\text{brub}}$, $f_{\mathrm{CO}}$, and $f_{{\mathrm{COO}}_{2}}$ are described in Table 2, and the $\mu_{a,\mathrm{Hb}}$, $\mu_{a,{\mathrm{HbO}}_{2}}$, $\mu_{a,\mathrm{brub}}$, $\mu_{a,\mathrm{CO}}$, and $\mu_{a,{\mathrm{COO}}_{2}}$ are the associated absorption coefficients. The absorption coefficients for melanin and hemoglobin were sourced from a database compiled by Jacques and Prahl.^25^ Bilirubin absorption coefficients were obtained from Bydlon et al.,^26^ and cytochrome C oxidase absorption coefficients were obtained from Mason et al.^27^ The scattering coefficient is the same for both the epidermis and dermis and is calculated using the following expression:^21^

$$\mu_{s}^{\prime }=a[f_{\text{Ray}}{\left(\frac{\lambda }{500\;\mathrm{nm}}\right)}^{-4}+(1-f_{\text{Ray}}){\left(\frac{\lambda }{500\;\mathrm{nm}}\right)}^{-b}].$$ (5)

To calculate the refractive index n, we use the following equation:^5^^,^^21^^,^^28^

$$n=1.309-4.346\cdot 10^{2}\lambda^{-2}+1.6065\cdot 10^{9}\lambda^{-4}-1.2811\cdot 10^{14}\lambda^{-6}.$$ (6)

Finally, the expression to calculate the anisotropy factor g is given in the following equation:^21^^,^^29^

$$g=0.62+29\;{\mathrm{nm}}^{-1}\cdot 10^{-5}\lambda .$$ (7)

To simplify the fitting process, we set the thickness of the epidermis $d_{\mathrm{epi}}$, the dermis $d_{\mathrm{der}}$, the fraction of Rayleigh scattered light $f_{\text{Ray}}$, and the Mie scattering power b to constant values, as shown in Table 1. Other parameters that will be predicted together with their description and boundary values are shown in Table 2.

**Table 1:** Values of fixed model parameters.

Parameter	b (−)	$f_{\text{Ray}}$ (−)	$d_{\mathrm{epi}}$ (cm)	$d_{\mathrm{der}}$ (cm)
Value	1.2^2^^,^^23^	$1\times 10^{-7}$ ^23^	0.01^5^^,^^30^	1^5^

**Table 2:** Description and boundary values of simulated model physiological parameters.

Parameter	Description	Minimum	Maximum
$f_{m}$ (−)	Volume fraction of melanin	0.001	0.05
$f_{\mathrm{Hb}}$ (−)	Volume fraction of deoxyhemoglobin	0.001	0.05
$f_{{\mathrm{HbO}}_{2}}$ (−)	Volume fraction of oxyhemoglobin	0.001	0.05
$f_{\mathrm{brub}}$ (mM)	Millimolar concentration of bilirubin	$1\times 10^{-7}$	0.1
$f_{\mathrm{CO}}$ (mM)	Millimolar concentrations of reduced cytochrome C oxidase	$1\times 10^{-7}$	2
$f_{{\mathrm{COO}}_{2}}$ (mM)	Millimolar concentration oxidized cytochrome C oxidase	$1\times 10^{-7}$	2
a ($\frac{1}{\mathrm{cm}}$)	Scattering coefficient at 500 nm	20	80

### 2.2 Spectra Simulation and *In Vivo* Data Acquisition

**Fig. 2:**
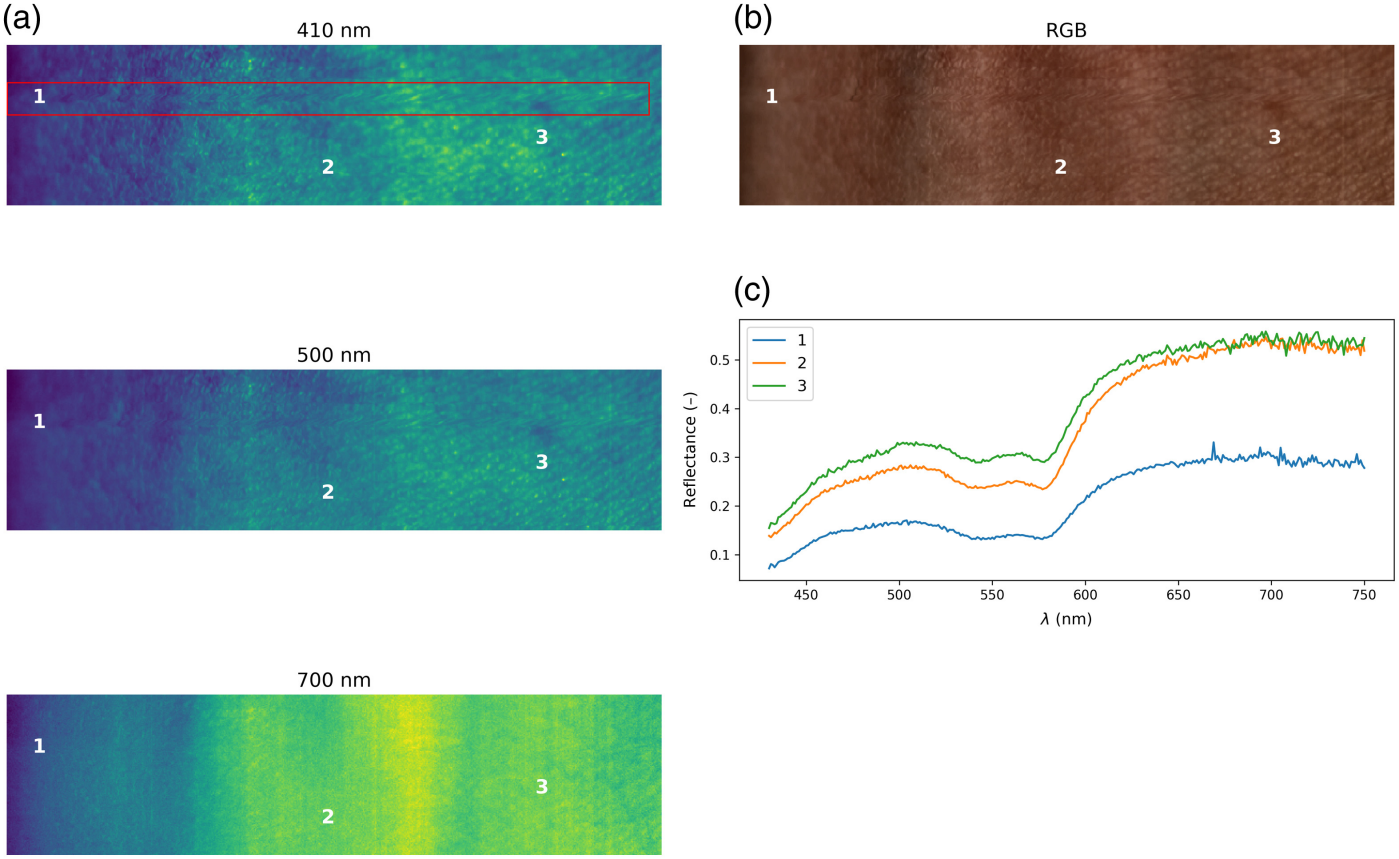
Three selected spectral bands (a). RGB image extracted from the hyperspectral image (b). Three selected measured reflectance spectra (c).

*In vivo* data were collected by acquiring an image of a forearm of 22 healthy volunteers of Caucasian skin types (Fitzpatrick types II to III) using a custom-built hyperspectral imaging system^31^ where the spectra from 21 volunteers were used to evaluate model accuracy and on one subject the cuff test was performed to additionally evaluate if the model detects a change in blood oxygenation during the experiment. All subjects gave informed consent. The experimental protocol was approved by the Slovenian National Medical Ethics Committee (0120-629/2016-3; KME 66/01/17) and conforms to the principles expressed in the Declaration of Helsinki. The original data were collected in the spectral range 400 to 1000 nm and were then normalized using the following equation:^1^

$$I_{\mathrm{ref}}=\frac{I_{\text{raw}}-I_{\text{dark}}}{I_{\text{white}}-I_{\text{dark}}},$$ (8)

where the measured $I_{\text{raw}}$ is the raw intensity; $I_{\text{dark}}$ is the dark current intensity, which was measured when the camera shutter was closed; and $I_{\text{white}}$ is the standard white reference intensity. Finally, the spectral range was reduced to 430 to 750 nm with a 1-nm step, resulting in 321 spectral bands. In Fig. 2, we show the three selected spectral bands with three reflectance measurements taken from different sites as well as the RGB reconstructed image. In addition, as the HSI system performs pushbroom scanning, visual artifacts can occur due to slight hand movements. This can be seen by observing the upper half of the spectral band at 410 nm, which is marked with a red box.

**Fig. 3:**
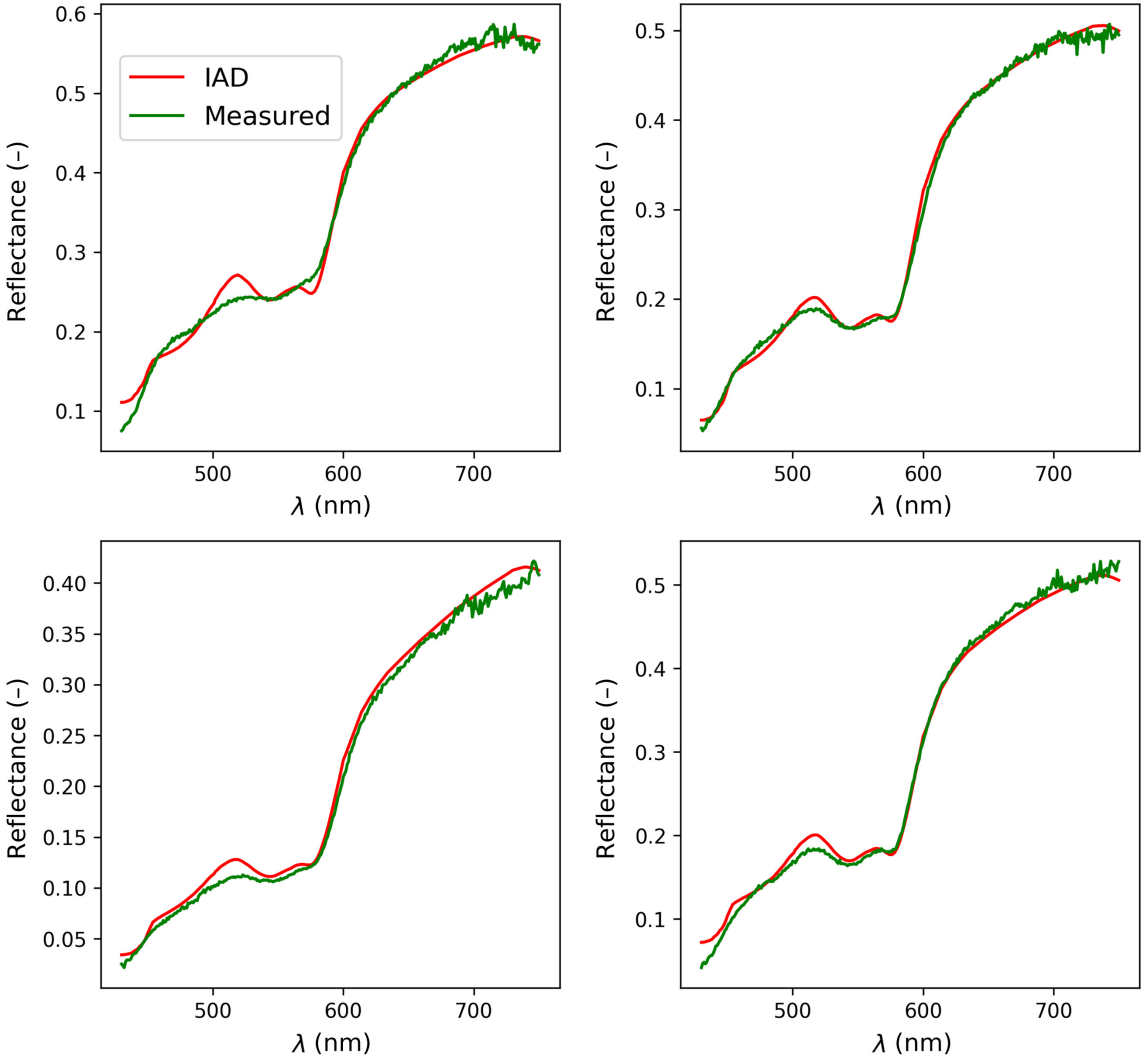
Measured spectra and their respective IAD-fitted spectra for four randomly selected instances.

To obtain the parameters from the observed spectra, IAD was used together with the model described in Sec. 2.1. The IAD algorithm starts from the initial estimate of the tissue parameters and then performs an iterative curve fitting to obtain the tissue parameters for which the fitted spectra are as close as possible to the measured ones.^21^ The Levenberg–Marquardt algorithm was used to perform the fitting, with the maximum number of iterations set to 200.^21^ In Fig. 3, we show four randomly selected measured spectra and their respective IAD-fitted counterparts. As can be seen in the figure, IAD yields a smooth spectrum, whereas the measured spectra contain noise, especially in the 650 to 750 nm range. As demonstrated in our previous work,^21^ the IAD algorithm is robust and accurate, having an accuracy within 2% of the gold standard IMC methods. Therefore, the fitted spectra agree well with the measured spectra because the IAD algorithm is capable of modeling all complexities of human skin. However, the parameter fitting problem has no unique solution and is non-linear, which makes it difficult to obtain the parameters directly from the spectra without imposing constraints, as already described in Sec. 1.

In this study, we assumed that training on *in vivo* data could potentially be expensive and would be highly dependent on the observed tissue. Therefore, we simulated 70,000 spectra using the model described in Sec. 2.1, of which one half was used for Bayesian inference, and the other half was used to train the F-NN. As the parameters described in Table 2 spanned different orders of magnitude, we normalized them in the range [0,1].

### 2.3 Proposed Model

**Fig. 4:**
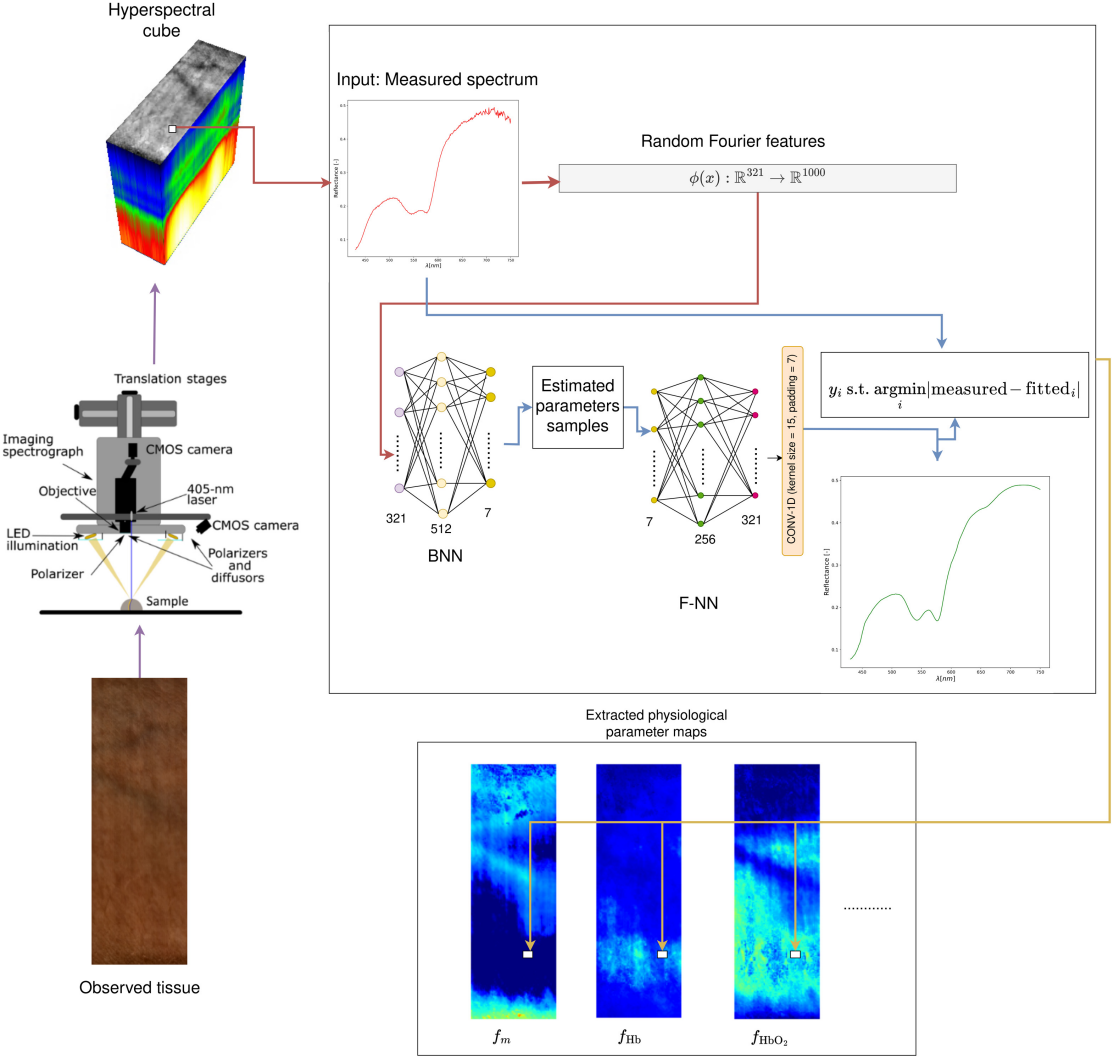
Proposed model architecture.

We consider the problem of direct extraction of physiological parameters from the diffuse reflectance spectra. As the measured diffuse reflectance spectra from which we want to extract tissue parameters are noisy, and there is no single best fit due to the ill-posedness of the IAD, we propose to model the relationship between the spectra and the parameters as a distribution instead of making a point estimate. The proposed model is shown in Fig. 4. As can be seen in the figure, the model consists of two main components. The first component is the Bayesian neural network, which takes the transformed input spectra and produces the instances of tissue parameters given the observed spectra. Next, F-NN is used to convert the sampled tissue parameters back into spectra and compare them with the measured spectra. The parameters for which the F-NN predicted spectra have the lowest mean absolute error (MAE) are used as output. In Sec. 2.3.2, we discuss all the decisions made to construct the BNN, whereas in Sec. 2.3.3, we discuss the parameterization of the F-NN model.

#### 2.3.1 Random Fourier features

We start this section with the assumption that the relationship between spectra and physiological parameters cannot be efficiently described as the linear combination of the reflectance values at specific wavelengths. Therefore, non-linear transformations have to be used. This can be done either directly, e.g., using the NNs, or implicitly, as it is used in Gaussian process regression or support vector machines (SVMs). Although the SVMs are used for HSI classification problems,^1^ support vector regression^32^ is almost never used for regression tasks for the extraction of physiological parameters from hyperspectral images. We hypothesize that the main reason for this is the poor scalability of those models on larger datasets.

By considering the good performance of kernel-based methods for HSI classification tasks and their poor scalability for larger datasets, we are interested in the mapping of the input spectra x in a high-dimensional space such that the inner product of transformed vectors approximates kernel function k

$$k(x_{i},x_{j})=\langle \psi (x_{i}),\psi (x_{j})\rangle \approx \phi (x_{i}{)}^{T}\phi (x_{j}).$$ (9)

One way to perform such non-linear transformation is RFFs.^20^ In this study, we approximated the Gaussian kernel using the following scheme:

$$\omega ∼N(0,I),\;b∼\text{Uniform}(0,2\pi ),\;\phi_{\omega }(x)=\sqrt{\frac{2}{D}}[\begin{array}{ccc} \cos(\omega_{1}^{T}x+b_{1}) & \ldots & \cos(\omega_{D}^{⊤}x+b_{D}) \end{array}{]}^{T},$$ (10)

where x is the spectrum, and ϕ(x) is the transformation function. As it can be seen from Eq. (10), $\phi (x_{i})$ can be obtained by concatenating $z_{\omega }(x)=\sqrt{2}\;\cos(\omega_{i}^{T}x+b_{i})$ D times and normalizing each component by $\sqrt{D}$.

#### 2.3.2 Bayesian neural network

When it comes to modeling the relation between the spectra and the tissue parameters, ANNs have been shown to outperform alternative models such as random forests (RFs) and generalized linear models.^12^ However, given that in practice, the observed spectrum can be noisy or affected by the use errors,^18^ and it is not guaranteed that the mentioned models will always produce plausible parameter estimates.

BNNs^33^^,^^34^ are an extension to ANNs in which the weights are treated as probability distributions, thus allowing us to obtain multiple predictions given the observed spectra. We start by defining model likelihood as a normal distribution having homoscedastic variance $\sigma^{2}$

$$P(y|x,W,\sigma^{2})=\overset{N}{\underset{i=1}{\prod }}N(f_{W}(\phi (x_{n})),\sigma^{2}I),$$ (11)

where x is the spectra, y is the physiological parameters, and W is the neural network weights. Next, for a dataset of spectra/parameters pairs D={x,y}, we are interested in obtaining the posterior

$$P(W,\sigma^{2}|D)=\frac{P(y|x,W,\sigma^{2})\cdot P(W)\cdot P(\sigma^{2})}{P(D)}.$$ (12)

Finally, we sample from the posterior predictive distribution to obtain the parameter candidates:

$$P(y_{\text{new}}|x_{\text{new}})=\int P(y_{\text{new}}|x_{\text{new}},W,\sigma^{2})\cdot P(W,\sigma^{2}|D)dW\;d\sigma^{2}.$$ (13)

When it comes to the network weights prior P(W), we chose normal distribution with fixed variance $P(W)=N(0,\sigma_{w}^{2}I)$ which is most commonly used in practice,^35^ and for the variance prior $P(\sigma^{2})$, we selected $\sigma^{2}∼\text{InverseGamma}(0.5,0.1)$, with a belief that small variance values are more likely while still allowing for the possibility of large variance values.

In this paper, we used a BNN consisting of a single hidden layer with 512 neurons and the ReLU activation function. To perform the Bayesian inference, we used black box variational inference (BBVI) implemented in Pyro^36^ with mean field Gaussian as the variational distribution. BBVI was performed with batch size 100 for 150 epochs using Adam optimizer, where for the first 100 epochs, the learning rate was set to $10^{-3}$, and for the last 50 epochs, the learning rate was lowered to $10^{-4}$. In addition, we used gradient clipping with a norm set to 5. Before making predictions, we sampled $500\;W,\sigma^{2}$ pairs, meaning that the 500 forward passes will be done for each spectrum $x_{\text{new}}$. Finally, although the range of the parameters is limited in the simulated dataset, there is no guarantee that the proposed BNN predictions will always be positive. Therefore, if negative parameters are inferred, we set them to zero in the postprocessing stage.

#### 2.3.3 F-NN

As the BNN outputs multiple solutions for a given spectrum, we are interested in building a model that can quickly verify how the parameters predicted by BNN fit the observed spectrum. Although the forward AD is much faster and less resource-intensive than the MC, it is still not suitable for near real-time use cases, especially for multi-layered tissue models. Based on the previous work, which had shown that ANNs^11^^,^^37^ achieve high accuracy as an approximator for a forward model, we used standard feed-forward ANN with one hidden layer. Therefore, to select the best solution from the candidate outputs by the RFF + BNN model, we trained an F-NN that maps predicted parameters and outputs the spectra. The network was trained on simulated data with the normalized tissue parameters as input and the spectra as output. This model is then used to select the parameters that result in the best-fitting spectra, i.e., to minimize the MAE between the measured and F-NN fitted spectra. As the forward problem is generally easier to solve, in contrast with the inverse mapping ML models and BNNs, we started building our model consisting of a single hidden layer of 256 neurons, ReLU activation, and an output layer having 321 neurons with the dropout with the probability of 0.2 between the hidden and output layer. However, we noticed that the F-NN model does not always produce smooth spectra, and therefore, we added an additional convolutional layer on top of the output layer. It consists of a single filter with a size of 15. This adjustment not only produced smoother spectra but also improved overall results when combined with RFF + BNN. All other hyperparameters (number of epochs, batch size, and optimizer) were used as in Sec. 2.3.2. In cases where the tissue model is much simpler and can be modeled using simple equations such as BL law,^19^ we argue there is no need to train an F-NN.

## 3 Experiments and Discussion

### 3.1 Experiments on Simulated Data

In this section, we describe the experiments and discuss the simulated dataset. As already mentioned, this study assumes that the real data will not be used in a training process. In contrast with our previous study,^17^ here, we assume that the range for most of the real parameters is known and that the surfaces are mostly flat, i.e., cosine of the surface inclination angle is close to 1. These assumptions about the real data simplify the problem, making it easier to achieve good predictive performance by uniformly sampling simulated parameters. We evaluate parameter estimation performance using MAE, which is more robust to outliers than the root mean squared error.

The first step in the process of obtaining acceptable results is to select the appropriate model architecture for mapping the observed spectra to parameter values. Currently, the baseline architecture for parameter estimation from raw spectra is based on ANNs.^12^^,^^14^ We also consider a much simpler ridge regression model as well as the more complex 1D-CNN model.^38^ In addition, we test the performance of the RFF-transformed input on ANN and ridge regression.

For ANNs, we selected an architecture consisting of a single hidden layer of 512 neurons with a ReLU activation function. We additionally set the dropout with a probability of 0.2 for regularization during training. 1D-CNN consists of three convolutional layers with 64 filters of size 3 with max-pooling of size 2 applied after each convolutional layer. Finally, features from the convolutional layers are passed to the fully connected layer of dimension 512. All neural network models (ANN, RFF-NN, and 1D-CNN) were trained by minimizing the mean squared error use. Similarly to the BNN inference already described in Sec. 2.3.2, we used the Adam optimizer and set the number of epochs to 150 where for the first 100 epochs, we trained the model using the learning rate 0.001, and for the last 50 epochs, we lowered the learning rate to 0.0001. The batch size for all NN-based models was set to 100. For the ridge regression model, we set the regularization parameter α to 1, and the solution was obtained analytically. Finally, we set the dimensionality of RFF to 1000. All hyperparameter values were selected based on previous experience and trial and error approach because an extensive grid search would be too costly to perform.

**Fig. 5:**
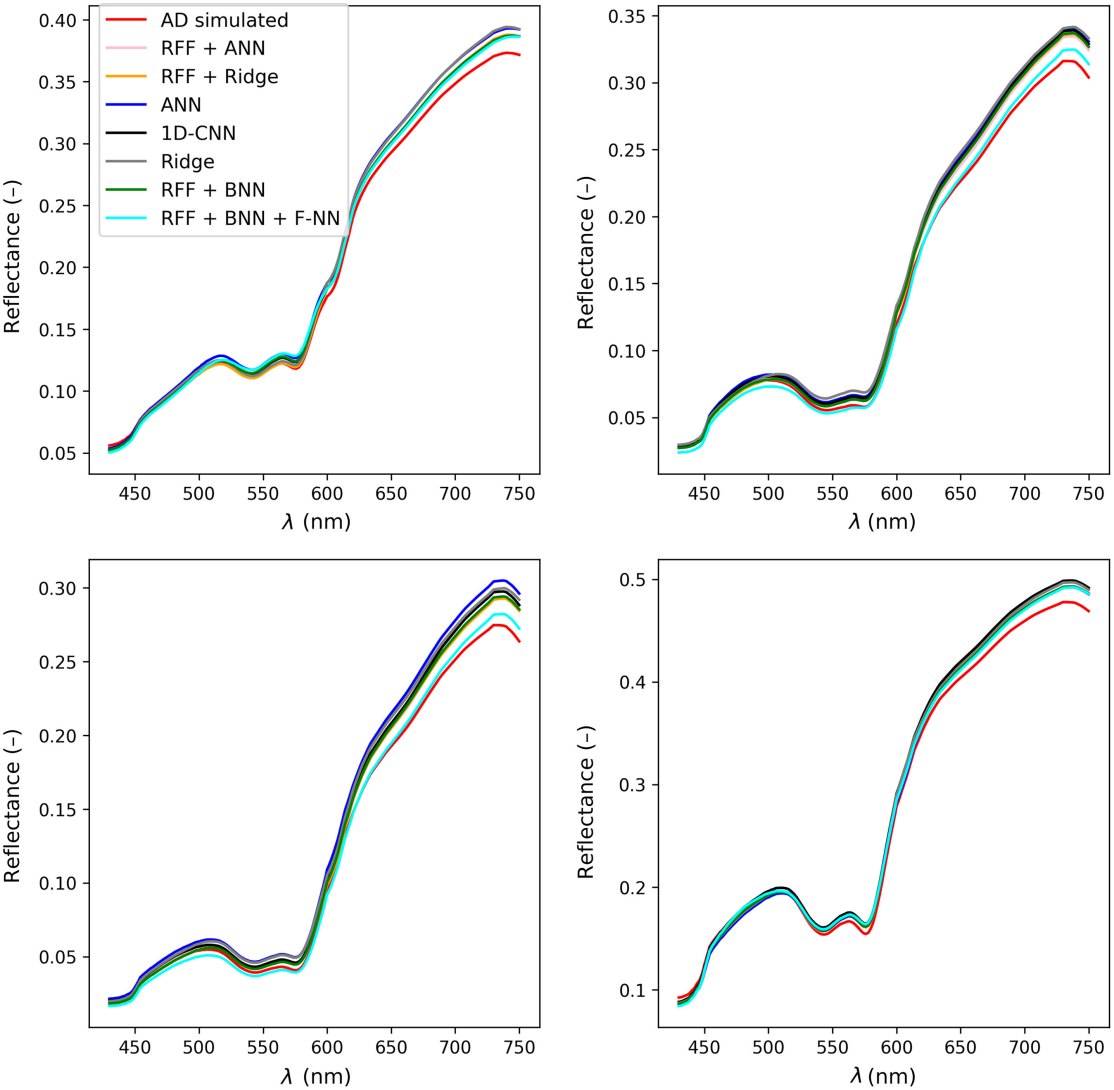
Four randomly selected AD simulated spectra and their respective forward-fitted spectra from ML models.

In Table 3, we show the results of the 10-fold cross-validation using the simulated data. As can be seen, all models perform well when tested on the simulated spectra without noise. ANN with RFF transformed spectra (RFF + ANN) has the best performance while having less trainable parameters than 1D-CNN. Also, it is interesting that the ridge regression models also perform relatively well considering their simplicity. Taking these results into consideration, we select the RFF-based ANN architecture as a baseline for building the BNN, whose performance is also shown in Table 3. To obtain better insights into the overall fitting performance, we use the AD to fit the spectra from the predicted parameters, which is shown in Table 4. As can be seen from the table, all models achieve acceptable performance on clean simulated spectra. To better visualize the results, we show the two randomly selected AD simulated spectra and their corresponding spectra predicted by the ML models in Fig. 5, where it can be seen that the parameters from all models agree well with the simulated spectra.

**Table 3:** Comparison of the model MAE for a specific parameter on the simulated dataset.

Parameter	Ridge	RFF + ridge	RFF + ANN	ANN	CNN	RFF + BNN	RFF + BNN + F-NN
$f_{m}$	0.00213	0.00096	**0.00032**	0.00105	0.00051	0.00059	0.0012
$f_{\mathrm{Hb}}$	0.00287	0.00189	**0.00036**	0.00103	0.00065	0.00084	0.0017
$f_{{\mathrm{HbO}}_{2}}$	0.00392	0.00256	**0.00042**	0.00119	0.00067	0.00119	0.00283
$f_{\text{brub}}$	0.00994	0.0079	**0.00096**	0.00255	0.00141	0.00366	0.00681
$f_{\mathrm{CO}}$	0.1585	0.11753	**0.01781**	0.04774	0.0275	0.05365	0.11081
$f_{{\mathrm{COO}}_{2}}$	0.19666	0.19413	**0.02096**	0.07619	0.02858	0.10858	0.1875
a	2.38826	1.35068	**0.39932**	1.05516	0.65191	0.68795	1.51752

Note: The best results are highlighted in bold.

**Table 4:** Comparison of the forward fitting model MAE on simulated spectra.

Model	MAE
**RFF + BNN + F-NN**	**0.0077**
RFF + ANN	0.0082
RFF + BNN	0.0086
1D-CNN	0.0086
RFF + ridge	0.0089
ANN	0.0094
Ridge	0.0114

Note: The best results are highlighted in bold.

**Fig. 6:**
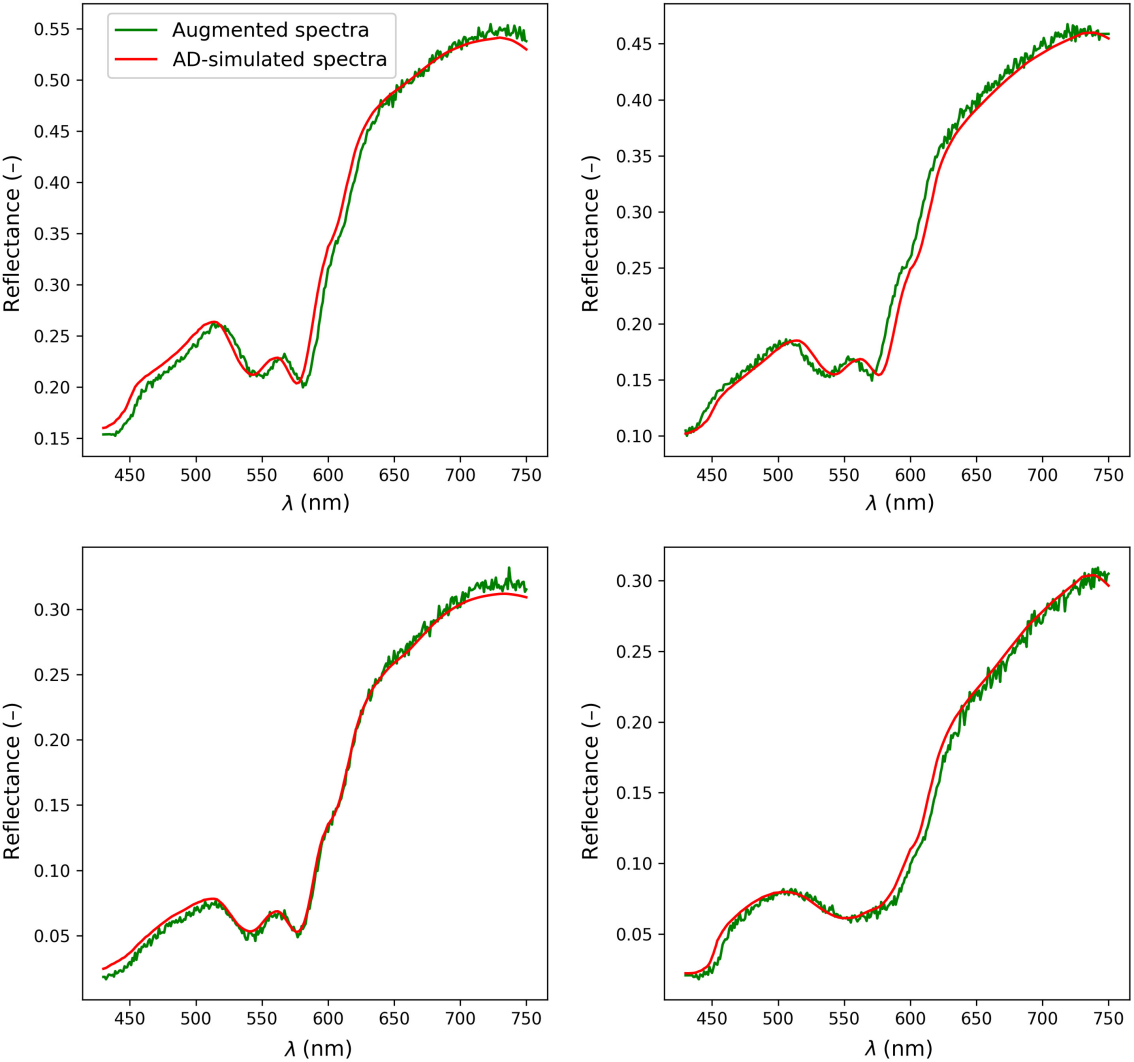
Four randomly selected simulated signals with their respective augmented versions.

Next, we test the robustness of all models by augmenting the spectra based on the recent work by Scarbrough et al.^18^ where it was shown that the ML models were generally more robust to Gaussian noise but less robust to signal scaling and rotation. Therefore, we augment the AD simulated spectra by rotating each spectrum randomly by 5% on the tails, randomly shifting the spectra for ±10-nm wavelengths and adding Gaussian noise with mean 0 and standard deviation 0.002 in the spectral range of 430 to 630 nm and Gaussian noise with mean 0 and standard deviation 0.008 for the 631 to 750 range. We show an example of a randomly selected augmented signal in Fig. 6. Although the augmented and simulated spectra look similar to Fig. 3, it is still not guaranteed that all use errors will be covered by this experiment. Therefore, we trained all models on the simulated spectra without augmentations and validated the performance on the augmented spectra. As this problem is ill-posed, we are mainly interested in an overall fitting performance, which is done using the ML estimated parameters, fitting the spectra using AD and observing the overall MAE between the fitted and augmented spectra. In addition, we use the F-NN model to fit the spectra to compare the forward models. In Table 5, we show the results of the 10-fold cross-validation for a specific model. As was expected, when the augmentations are applied to the spectra, differences among the results for different models become more obvious. Finally, this experiment shows that there is a clear benefit of using BNNs as the RFF + BNN model with F-NN selection obtained the best results.

**Table 5:** Comparison of the forward fitting model MAE on augmented simulated spectra.

Model	MAE (AD)	MAE (F-NN)
**RFF + BNN + F-NN**	**0.0107**	**0.0072**
RFF + BNN	0.0131	0.0115
RFF + ridge	0.0162	0.0149
RFF + ANN	0.0178	0.0176
1D-CNN	0.0187	0.0182
ANN	0.0201	0.0201
Ridge	0.0234	0.0217

Note: The best results are highlighted in bold.

### 3.2 Experiments on *In Vivo* Data

In this section, we show and discuss the results of the *in vivo* data. As shown in Fig. 3, measured spectra contain noise, especially for wavelengths above 600 nm. Also, due to the ill-posed nature of the problem, there is not a unique solution, and therefore, it is not enough to just compare the ML models against the IAD (Table 6), but it is also required to observe the forward-fitted spectra and compare them against the measured spectra (Table 7). Therefore, we analyze the results by looking at both tables simultaneously. In addition, we are particularly interested in $f_{m}$, $f_{\mathrm{Hb}}$, $f_{{\mathrm{HbO}}_{2}}$, and $f_{\text{brub}}$ due to their importance for diagnostic purposes.

**Table 6:** Comparison of the models’ MAE for tissue parameters extracted from *in vivo* spectra. The best and second-best results for each parameter are bold and italics, respectively.

Model	$f_{m}$	$f_{\mathrm{Hb}}$	$f_{{\mathrm{HbO}}_{2}}$	$f_{\text{brub}}$	$f_{\mathrm{CO}}$	$f_{{\mathrm{COO}}_{2}}$	a
ANN	0.009815	0.006140	*0.006801*	0.052051	0.120164	*0.244695*	9.257428
RFF + BNN	*0.005883*	0.005906	0.008436	*0.026313*	0.143875	0.275963	*8.172330*
RFF + BNN + F-NN	**0.004752**	**0.004616**	0.007104	**0.024946**	0.133573	0.291012	**7.777429**
1D-CNN	0.010523	0.008642	**0.005404**	0.064111	**0.103635**	0.502473	8.780724
RFF + ANN	0.007106	0.006245	0.006989	0.036719	0.119059	**0.235668**	8.532389
RFF + ridge	0.007941	*0.005507*	0.010006	0.036934	*0.107643*	0.372919	8.550158
Ridge	0.006890	0.010886	0.009563	0.050608	0.110290	0.541280	12.862863

**Table 7:** Overall fitting performance on the *in vivo* spectra.

Model	MAE
IAD	0.014933
**RFF + BNN + F-NN**	**0.014107**
RFF + BNN	0.019810
RFF + ANN	0.022633
RFF + ridge	0.024262
1D-CNN	0.028409
ANN	0.030921
Ridge	0.053462

Note: The best results are highlighted in bold.

For better visibility, in Table 6, we highlighted the best results with bold and the second-best results with italics. If we compare the results on measured data with the results on the simulated data from Table 3, it can be seen that the models perform much better on clean simulated data, whereas using augmentations in validation is more realistic and closer to the results on *in vivo* spectra. These results are also supported by the recent study by Scarbrough et al.^18^ Moreover, although the classic NN models outperform the BNN-based models on simulated spectra, they are still less robust when tested on *in vivo* spectra.

We first observe and analyze the results of commonly used algorithms trained on raw simulated spectra, namely, ridge regression, ANN, and 1D-CNN. Ridge regression is obviously the simplest model, and although it estimates $f_{m}$ relatively close to the IAD, all other parameters are far away from the IAD, resulting in bad forward-fitted spectra, as it can be seen in Table 7. Next, the ANN and 1D-CNN models have better results but also fail to achieve acceptable overall performance. We believe that the main reason for such low overall performance lies in bad MAE for $f_{m}$. Importantly, transforming the data using RFFs improves the overall results. This can be confirmed by looking at the good performance on $f_{{\mathrm{COO}}_{2}}$ and a, which are important for good overall fitting, especially for the wavelengths larger than 650 nm. RFF + BNN + F-NN produces results that are closest to the IAD for four out of seven parameters while also having other parameters close to the best result. Finally, all BNN models achieve a high MAE for $f_{{\mathrm{HbO}}_{2}}$, which is not the case for 1D-CNN. We argue that the main reason for this is that the 1D-CNN is better at detecting changes in the signal shape for the 500 to 600 nm range. In the future, ML models could possibly be improved either by building a hybrid model where both RFF + ANN and 1D-CNN were combined or by considering only the 500- to 600-nm range for predicting the $f_{{\mathrm{HbO}}_{2}}$.

**Fig. 7:**
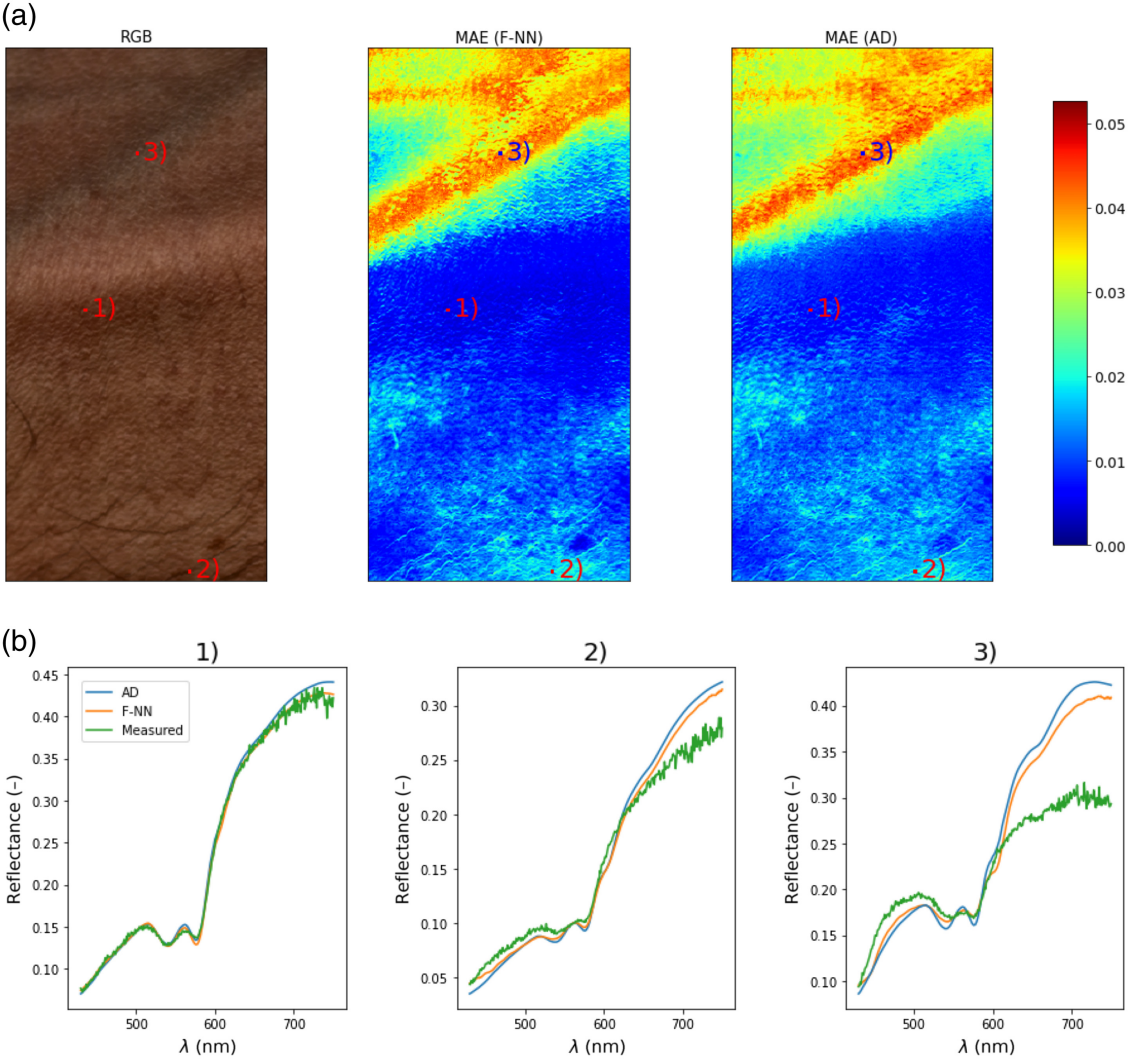
(a) Spatial distribution of errors between measured spectra and spectra estimated using AD or predicted using F-NN from the parameters predicted by the RFF + BNN + F-NN model. (b) Three selected measured spectra with their AD and F-NN estimated spectra from (1) normal skin, (2) skin containing hairs, and (3) blood vessels.

Next, Fig. 7 shows the spatial distribution of MAE between fitted and measured spectra. This was done first by estimating the parameters using the RFF + BNN + F-NN model and then using the predicted parameters to estimate the spectra using AD and F-NN separately. The main goal of this experiment was to obtain a better insight into the tissue regions where the model performs poorly, but also to visually compare the difference between AD and F-NN forward models. As can be seen from the figure, both F-NN and AD predict roughly the same regions with high errors (MAE>0.03). In addition, we show the three randomly selected spectra with the spectra estimated using F-NN and AD. As can be seen from the results, the models perform poorly in the regions containing blood vessels. This behavior is expected because the spectra from the blood vessels require increasing the intervals of certain parameters and possibly introducing some parameters that were currently fixed (i.e., b). However, we believe that this would possibly degrade the predictive performance for the parts of the skin where the model performs well.

Next, we performed a cuff test on a healthy volunteer, where a blood pressure cuff was placed on the upper arm and inflated to 200 mmHg for ∼3  min. During this experiment, three images were taken: before, during, and after the cuff test with the goal of estimating blood oxygenation using the estimated parameters from the tissue model shown in Fig. 1, using the following equations:

$${\mathrm{StO}}_{2}(\%)=100\%\cdot \frac{f_{{\mathrm{HbO}}_{2}}}{f_{\mathrm{Hb}}+f_{{\mathrm{HbO}}_{2}}}.$$ (14)

**Fig. 8:**
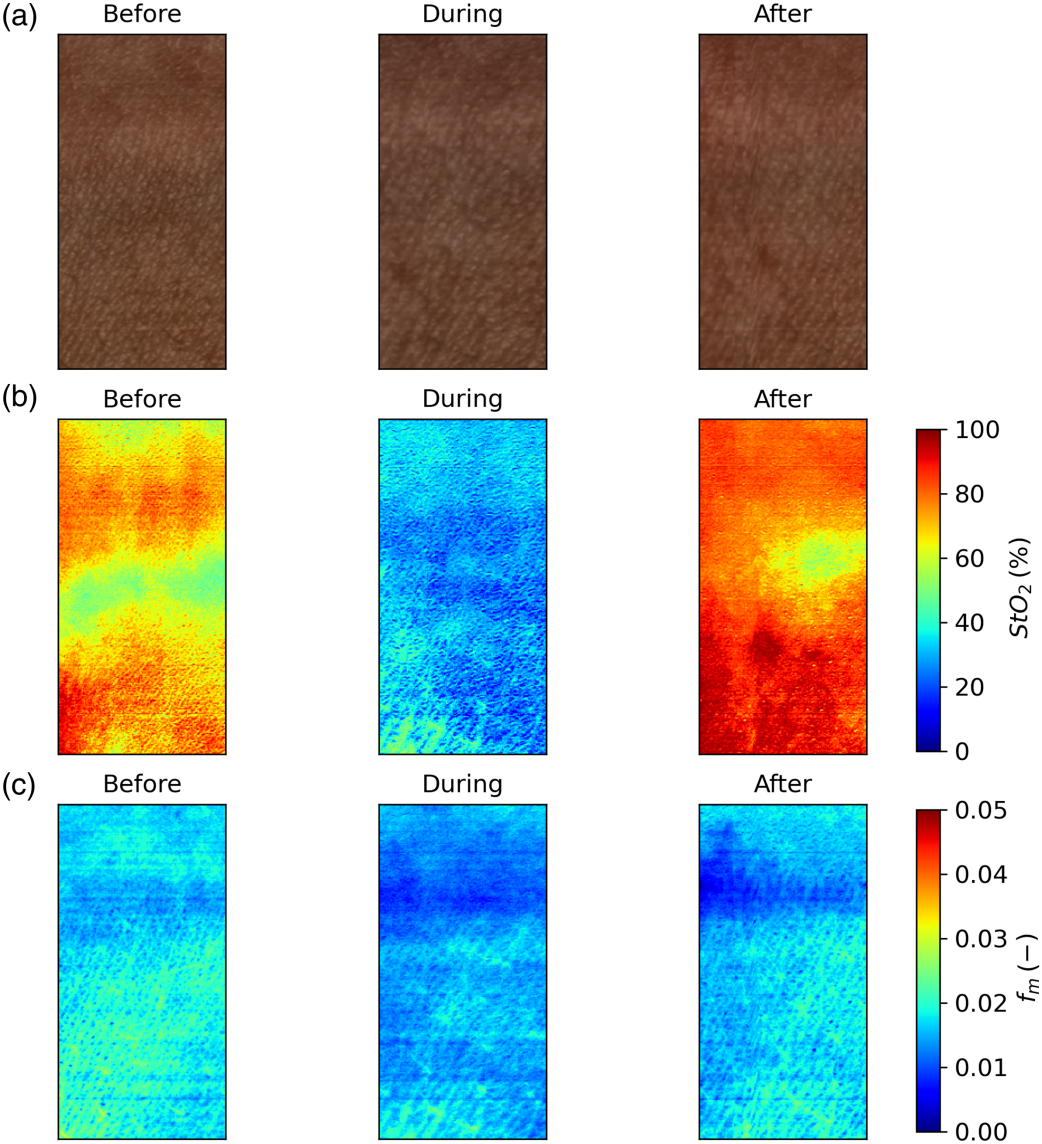
(a) Reconstructed RGB images. (b) RFF + BNN + F-NN estimated ${\mathrm{StO}}_{2}$. (c) RFF + BNN + F-NN estimated $f_{m}$.

As it can be seen in Fig. 8, the model detects changes in the ${\mathrm{StO}}_{2}$ during the cuff test, whereas the estimated $f_{m}$ is relatively stable.

Finally, we present the estimation speed of the models in Table 8. Model performance was estimated on a workstation consisting of two Intel^®^ Xeon^®^ Processors E5-2620 v4 CPUs, 128 GB RAM, and three GeForce RTX 2080 Ti graphics cards. Before the test, all spectra were loaded into the memory, and preprocessing was not taken into consideration when measuring time. RFF + BNN and NN models were run on GPU, whereas the ridge regression and RFF were calculated on CPU. As can be seen, the RFF + BNN model has a much lower estimation time in comparison with the IAD. However, it is also not as fast as the NN-based models because it has to perform multiple forward passes and select the best fit using F-NN. During the measurements, we identified that the multiple forward passes took roughly 80% of the time, meaning that the performance can be possibly improved by considering a smaller model and performing the multiple forward passes in parallel. In addition, as the RFF + BNN model outputs multiple predictions, meaning that multiple forward passes through the NN are required, a hyperspectral image with high spatial resolution could not be processed in a single step but requires spectra to be organized into multiple batches.

**Table 8:** Comparison of the time in microseconds required to estimate the parameters of a single spectrum: for IAD, RFF + BNN + F-NN, N-based models, and RFF + ridge model.

	IAD	RFF + BNN + F-NN	ANN–CNN	RFF + ridge
Time (μs)	$3.6\times 10^{5}$	$1.1\times 10^{3}$	**94 to 96**	140

Note: The best results are highlighted in bold.

The main limitation of this study is the relatively homogeneous cohort consisting of 22 healthy volunteers with Fitzpatrick skin types II to III. Although the cuff test was used to evaluate the changes in tissue properties, it is not guaranteed that the model will correctly predict abnormal physical states such as dermatitis or melanoma. However, we argue that it is possible that such abnormalities will have a large F-NN approximation error, similar to those shown in Fig. 7.

Finally, in our previous study,^17^ one of the limitations was the usage of the Gaussian mixture model (GMM) to sample realistic parameter combinations that will then be used to simulate spectra using AD. Using this approach, it was necessary to have at least one IAD-fitted image on which the GMM could be trained. As the surface topography and the common use errors described in previous chapters could affect IAD performance, there is no guarantee that all GMM sampled parameters would be realistic. Therefore, to avoid this limitation, it is better to build a simulated dataset based on uniform sampling but with a much smaller range for specific parameters. In other words, sampling the $f_{\mathrm{Hb}}$ and $f_{{\mathrm{HbO}}_{2}}$ in the range of [0.001, 0.05] rather than [0.001, 0.1] resulted in a better performance and removed the need to use GMM-based sampling.

## 4 Conclusion and Future Work

In this study, we proposed a Bayesian neural network to solve the problem of direct extraction of physiological parameters from the diffuse reflectance spectra. All models were trained on the simulated spectra and evaluated on clean simulated spectra, augmented simulated spectra, and *in vivo* spectra obtained from the forearms of 22 healthy volunteers where the images from 21 volunteers were used to evaluate models and on one healthy volunteer a cuff test was performed to observe if the proposed model detects changes in blood oxygenation. The results have shown that using the RFF-transformed spectra generally improves the fitting results, whereas using the BNN with the F-NN to select the best fitting spectra leads to results that generally provide almost the same fitting results as IAD.

Although the results on the simulated data were generally acceptable for almost all classic ML models, the performance generally deteriorated when applied to the measured data, which can generally contain noise and use errors already described in the previous chapters. Therefore, we argue that it is not sufficient to test the models only on raw simulated spectra but is also necessary to test the robustness of the model, at least on the augmented simulated spectra, if the *in vivo* data are not currently available. Furthermore, as the problem is ill-posed in general, we argue that the overall fitting performance should be evaluated together with the performance for a particular parameter.

Although the results look promising, there are several directions that could be pursued to improve the results even further. First, as the hyperspectral images are larger than the regular RGB images, and the RFF-transformed spectra are even larger than the original HSI dimensionality, one would need to experiment with dimensionality reduction techniques, such as principal component analysis, to reduce the hyperspectral image size. Moreover, we believe that model performance could be further improved if the patches or whole images were considered as ML model input. As acquiring a large dataset of hyperspectral images is difficult, it would be necessary to propose a method to simulate patches or even whole tissues. Nevertheless, this work lays the foundation for future research on the application of simulation-based inference^39^ to biomedical HSI.

## Data Availability

The code and data are not publicly available at the moment but may be obtained from the authors upon reasonable request.
